# Resident interactions when affirming and correcting peers in a therapeutic community for women

**DOI:** 10.1108/tc-03-2021-0007

**Published:** 2021-11-26

**Authors:** Keith Leverett Warren, Nathan Doogan, Uwe Wernekinck, Fiona Claire Doherty

**Affiliations:** College of Social Work, The Ohio State University, Columbus, Ohio, USA.; College of Social Work, The Ohio State University, Columbus, Ohio, USA; Ohio Colleges of Medicine Government Resources Center, Columbus, Ohio, USA.; College of Social Work, The Ohio State University, Columbus, Ohio, USA.; College of Social Work, The Ohio State University, Columbus, Ohio, USA.

**Keywords:** Therapeutic communities, TC practice, Substance dependence, Prisons, Social network analysis

## Abstract

**Purpose –:**

While recent years have seen a number of studies of social networks in therapeutic communities (TCs) and other residential settings, these have primarily focused on male residents. This paper aims to conduct a longitudinal social network analysis of interpersonal interactions in a TC for women.

**Design/methodology/approach –:**

The data consists of a longitudinal directed social network of instances of feedback between 56 residents of a 16 bed TC for women over a period of 611 days. Mean age of the participants was 33.1 years, mean length of stay was 133.9 days and 91% of the participants were female. Feedback consisted of written affirmations for prosocial behavior and written corrections for contravening TC norms. Data was analyzed using a latent factor longitudinal social network model.

**Findings –:**

Residents react to peer intervention in complex ways. Residents reciprocated affirmations (B = 0.14, 95% confidence interval = 0.10, 0.18) and corrections (B = 0.20, 95% CI = 0.13, 0.25). Controlling for reciprocity, participants who received affirmations were more likely to affirm and correct peers (B = 0.10, 95% CI = 0.06, 0.15; B = 0.17, 95% CI = 0.10, 0.23), suggesting that the encouragement offered by affirmations leads to increased activity. Homophily by admission time occurred in both affirmations and corrections (B = 0.23, 95% CI = 0.10, 0.37; B = 0.51, 95% CI = 0.29, 0.74).

**Originality/value –:**

While affirmations and corrections serve as vehicles for behavioral reinforcement and social learning, they also allow residents to interact in ways that strengthen social bonds.

## Introduction

Researchers have begun to use social network theory and analysis as a framework for understanding processes in recovery from substance abuse (Best and Lubman, 2017; Best et al., 2015; Elreda et al., 2016; Jason, Light et al., 2014). This interest has extended to therapeutic communities (TCs), residential treatment programs for substance abuse in which mutual aid between peers is the primary method of clinical intervention (De Leon, 2000). Best et al. (2014) map the identification of TC residents with various social groups, including the TC itself. In a cross-sectional social network study of a 20-bed TC, Kreager et al. (2018) found evidence of homophily by race and admission time in resident interactions. The study also found transitivity (the tendency of two individuals who are connected to mutually connect with a third) in networks of peer corrections and some correlation between the friendship network and whether peers were willing to affirm or correct each other.

Given the clinical assumption that residents will interact to create a stable environment conducive to recovery, studies of cooperation among TC residents are of particular interest. In longitudinal analyzes of affirmations and corrections exchanged between men in three TCs, Doogan and Warren (2017a, 2017b) found a complex pattern that included reciprocity (when Resident A affirms Resident B and Resident B subsequently affirms Resident A), generalized reciprocity (when Resident A affirms Resident B and Resident B becomes more likely to affirm another peer) and a tendency for residents in the same entry cohort to preferentially interact (homophily by admission time).

Network studies of TCs have focused on male units, but multiple authors suggest that women may relate to peers in TC treatment differently than men do. In a study of a mixed-gender TC, Possick and Itzick (2018) find that women have complex attitudes toward female peers, on the one hand showing a desire for a family and intimate friendship and on the other frequently finding that desire to be frustrated. Eliason (2006) argues that the TC emphasis on relating to the entire community of 25 or more residents may be inappropriate for women, whose friendship networks are typically based on a small number of intimate friends. On the other hand, Mosher and Phillips (2006) report that women show concern for the entire community.

Literature on cooperation and social network structure also finds differences based on gender. There is evidence from economic studies that women are more altruistic than men – for instance, they typically give more to charity (Breeze and Thornton, 2006; Piper and Schnepf, 2008). There is evidence that women, in general, organize social networks somewhat differently than men. In a study of an online gaming society, Szell and Thurner (2013) found higher levels of reciprocity and clustering, and overall more network activity, among female participants. In a study of proximity, phone and face-to-face contacts, Psylla et al. (2017) also found that women were more active, in addition to forming more reciprocal dyads while spreading contacts more evenly. But the structure of social networks varies depending on the setting (Psylla et al., 2017; Yang et al., 2019); these studies might offer only weak guidance to the way in which social networks might develop in a TC. In this study, we analyze two social networks from a women’s TC unit. While the analysis is exploratory (we do not test formal hypotheses), we use similar previous studies of male TC units (Doogan and Warren, 2017a, 2017b) as a basis for comparison, which may elucidate differences in the interaction dynamics of exclusively male and exclusively female TC units. Knowledge of such differences may support the refinement of TC practice with respect to gender.

## Methods

### Data

As part of the mutual aid-based structure of TCs, residents affirm peers for prosocial actions and correct peers for actions that contravene TC norms (De Leon, 2000). Data for this study consists of archival records of 6,108 affirmations and 1,945 corrections exchanged between 56 female residents of a 16-bed gender-segregated TC unit over a period of 611 days. The records were kept for purposes of monitoring the clinical functioning of the facility. The TC was a freestanding minimum security correctional facility that drew residents from rural and suburban counties. Residents initially wrote each affirmation or correction on a form that included the name of the sender, the name of the receiver, the date and the content. Affirmations involved feedback for prosocial actions such as doing an unusually good job at a work position, helping a junior peer adjust to the TC or offering helpful comments during group. Corrections involved feedback for actions violating TC norms of behavior, which might include lying, making noise after lights out or speaking during a time when silence was mandated. The forms were turned into staff and evaluated for legitimacy by a committee of staff members and senior residents. For instance, if a resident affirmed a peer for good job performance the committee would make sure that the job had actually been done. After the affirmation or correction was determined to be legitimate, it was read in front of the community. It was then entered into a computer database. The affirmations and corrections form two longitudinal directed social networks, with an arrow going from each sender to each receiver and time resolution at a one-day period.

In addition to these records, the facility kept data on the entry time, age and race of residents. The facility also recorded residents’ scores on the level of service inventory-revised (LSI-R), a standardized instrument of recidivism risk factors that measure such variables as previous criminal history, employment and substance abuse history with a range of 0–54 (Andrews and Bonta, 1995). Mean age of the residents was 33.1 years (SD = 7.5), mean length of stay was 133.9 days (SD = 42.4) and the mean score on the LSI-R was 25.32 (SD = 6.05, minimum score = 13, maximum score = 40); risk of recidivism for these residents, therefore, ranged from low risk to moderate/high risk (Lowenkamp and Bechtel, 2001). In total, 91% of the women were white.

### Analysis

Our aim is to model the process by which the complex web of interactions between TC residents unfolds in time. In particular, we seek to learn how patterns among previously exchanged interactions affect which new interactions appear. To achieve this, we use a regression model in which whether one resident affirms or corrects another during a specific day is the dependent variable. Predictors of this interaction characterize specific patterns of the network’s history from the perspective of the individuals in a dyad. For example, for the A -> B dyad, it is possible to use the number of affirmations which A has previously *received* as a predictor of the probability that A might *give* affirmations. If A is more likely to give an affirmation after receiving one, she is paying help forward or demonstrating generalized reciprocity in the more standard evolutionary cooperation terminology (Pfeiffer et al., 2005). Dyads can also be characterized by exogenous factors describing the individuals or their relationship (e.g. race of sender; race of receiver; sender and receiver have the same race).

Standard regression models assume that each data point is independent of all others once we have conditioned on the appropriate control variables (Cranmer et al., 2017). This assumption is problematic when analyzing TC data, as a clinical change in TCs comes about through mutual influence (De Leon, 2000). The dependency of one resident’s actions on those of another is an assumption of the program. For instance, in this analysis, we assume that if A affirms or corrects B, it will influence the likelihood of B affirming or correcting other residents.

We, therefore, model the dependence of one resident’s actions on peers through the inclusion of variables that characterize the complex history of peer interactions. However, we only know the day on which each interaction occurred. We do not know the within-day timing of the interactions. Thus, if A affirms B one day and B affirms A the next day, we know which came first. But if A affirms B in the morning and B affirms A in the evening we do not know which came first. As a result, the considerable dependency between outcome observations is likely to remain even after controlling for all of the predictors on previous days.

We control for this nuisance dependency by including a latent factor component in our model. This added component models the remaining dependencies by assuming that correlation between observations can be characterized as a function of the closeness of two residents in a latent space; effectively, two residents who are more similar are closer in the space [A more comprehensive review of this type of method has been compiled by Kim et al., (2018)]. The latent factor component was added to a generalized linear mixed model (GLMM) using a probit link function. The GLMM also included three random effects. The first and second accommodated heterogeneity in the propensity for residents to be the (1) giver or (2) receiver of an interaction. The third accommodated heterogeneity in the number of interactions on each individual day. The latent factor component and the random effects components are used to cope with nuisance dependencies – we report them as part of the model but give minimal attention to the interpretation of those model components.

We are primarily interested in the temporal-structural dependencies represented in the coefficients of the predictors. For example, do residents tend to reciprocate affirmations on the days following the receipt of an affirmation? Do residents tend to correct individuals of a different race than their own?

### Measures

The model captures and summarizes the relationships between observed traits or previous events by residents (independent variables) and the occurrence of new interaction events (dependent variable). Many of the independent variables are, therefore, defined as network structures observed in previous time periods.

#### Dependent variable.

The presence of a directed tie (representing an affirmation or correction) from Resident A to Resident B. A tie can form either in reaction to the action of peers, due to individual characteristics or even some combination, though we do not investigate such combinations in this study.

#### Reciprocity.

If Resident A affirms or corrects Resident B, Resident B could be more likely to affirm or correct Resident A. Reciprocity is ubiquitous in social networks (Wasserman and Faust, 1994) and is fundamental to cooperation in groups (Axelrod, 1984). TC clinical theory states that residents are expected to engage in reciprocal helping behaviors, through which they learn the value of helping others (De Leon, 2000; Frye, 1984; Stevens, 2013).

Triad closure occurs when Resident A, Resident B and Resident C all affirm each other in some combination. Like reciprocity, triad closure is ubiquitous in social networks (Wasserman and Faust, 1994). Triad closure is also of interest in the context of TCs because there is evidence that social networks characterized by closed triads exert more influence on their members and are seen as more supportive (Centola, 2010; Wellman and Frank, 2001). Likely because of this, social networks characterized by closed triads have been found to influence resident outcomes following termination from the TC (Campbell et al., 2019; Warren et al., 2020). In the current analysis, this implies that each of the three residents knows that the others have exchanged affirmations or corrections, not an unreasonable assumption is given the small size of the unit and the public announcement of peer feedback. In a directed social network two types of triad closure are possible, transitivity and three cycles:

#### Transitivity.

If Resident A affirms/corrects Resident B and Resident B affirms/corrects Resident C, then Resident A will be more likely to affirm/correct Resident C, producing a transitive triad (Wasserman and Faust, 1994).

#### Three cycle.

If Resident A affirms/corrects Resident B and Resident B affirms/corrects Resident C, then Resident C is more likely to affirm/correct Resident A (Wasserman and Faust, 1994).

When a resident receives an affirmation/correction, she might become more likely either to send or receive a further affirmation/correction:

#### Indegree activity.

If Resident A receives an affirmation/correction, she is more/less likely to send an affirmation/correction. If she is more likely to send one, this is a form of generalized reciprocity, in which residents receive help from one peer and pass it forward to another (Pfeiffer et al., 2005; Simpson et al., 2018; Stanca, 2009). In TC clinical literature, De Leon (2000) argues specifically that receiving affirmations can act to energize residents, increasing their activity, suggesting that affirmations might serve as a form of encouragement or emotional social support.

#### Indegree popularity.

If Resident A receives an affirmation/correction, she is more/less likely to receive another. In network terms, this is a form of preferential attachment (Barabási and Albert, 1999) and might indicate that affirmations and corrections alter the reputation of those who receive them (Rand and Nowak, 2013).

A TC resident might also change in response to her own activity. For instance, affirming a peer might make it easier to affirm another peer. Three variables assess this possibility:

#### Outdegree activity.

If Resident A sends an affirmation/correction, she is more/less likely to send a further affirmation/correction. If a resident is more likely to send a correction after sending an affirmation or vice versa, this suggests that she is trying to balance her effort and reputation in the TC (De Leon, 2000).

#### Out isolation activity.

This is the low end of outdegree activity, coded as one if the resident has not sent an affirmation at all and zero if she has. Out isolation activity tests whether residents who are not participating in the peer affirmation/correction system previous to a given time are more or less likely to begin participating.

#### Same peer concentration.

Measures the likelihood that, if Resident A has sent an affirmation/correction to Resident B, she will send an affirmation/correction to the same resident. TC clinical literature (De Leon, 2000) suggests that residents should be extending these behaviors to peers across the TC rather than concentrating them on one or a small number of peers. Residents might also affirm a peer to counterbalance a correction (De Leon, 2000), in which case we would see the same peer concentration of affirmations following corrections.

#### Time in therapeutic community activity and popularity.

The likelihood that residents will send or receive more affirmations/corrections depends on how long they have been in the TC. In this analysis, the periods tested are less than one month in the TC and more than four months in the TC, with the period between one and four months as the reference category. TC clinical literature suggests that residents should send fewer affirmations and corrections in their first month in the TC, as they are still learning about the program. On the other hand, it is also possible that residents will begin to detach from the TC when the end of treatment is in sight.

#### Attribute activity and popularity.

Likelihood of residents sending/receiving more or fewer affirmations/corrections depending on their age, LSI-R score and race.

#### Homophily variables.

Likelihood of residents exchanging more affirmations/corrections with peers of the same race, similar age, similar LSI-R or similar arrival time.

#### Head-turning activity.

In this unit, it was possible for residents to receive a correction for “head-turning,” defined as ignoring a peer behavior that should have been corrected. The idea of a sanction for refusing to sanction a peer is known from the evolutionary cooperation literature (Fowler, 2005). Receiving a head-turning correction is expected to lead to an increase in the number of corrections sent to peers.

#### Head-turning activity (whole unit).

Measures whether a correction for head-turning increases the activity of individuals who did not directly receive the correction.

#### Head-turning popularity.

It is possible that peers would respond to a head-turning correction by either affirming the individual who sent the head-turn correction or by correcting the individual. The latter might be an example of counter punishment (Nikiforakis, 2008).

#### Head-turning reciprocity.

Measures whether peers who receive a head-turning correction would reciprocate with an affirmation or correction.

#### Staff activity/popularity.

Likelihood of residents sending/receiving affirmations/corrections to/from peers after receiving an affirmation/correction from the staff compared with receiving one from a peer. For example, the staff head-turning activity coefficient represents the difference in residents’ response to a head-turn correction when it comes from a staff versus a peer. Thus, a zero staff head-turning coefficient suggests that residents do not react any differently when receiving a head-turn correction from staff or peers. Regardless of the size of the staff version of the coefficient, it must be interpreted in sum with the peer version to gain a sense for the absolute effect of a staff head-turn correction.

## Results

Results of the latent space models of interactions can be found in Table 1:

- *Affirmation reciprocity*: As expected, the women reciprocated affirmations (B = 0.14, 95% CI = 0.10, 0.18). However, they tended not to affirm peers who had recently corrected them (B = −0.09, 95% CI = −0.15, −0.04).
- *Transitivity and three-cycles*: There was no evidence of transitivity in either affirmation or corrections, but a tendency toward three cycles does occur across affirmations and corrections (B = 0.02, 95% CI = 0.00, 0.03).
- *Affirmation indegree activity*: Residents who received an affirmation were more likely to send both affirmations and corrections (B = 0.10, 95% CI = 0.06, 0.15; B = 0.17, 95% CI = 0.10, 0.23, respectively).
- *Affirmation indegree popularity*: Women who receive an affirmation are more likely to subsequently receive a correction from peers who did not give the affirmation (B = 0.14, 95% CI = 0.05, 0.22).
- *Affirmation outdegree activity*: Women who sent an affirmation were less likely to subsequently send an affirmation but more likely to subsequently send a correction (B = −0.07, 95% CI = −0.10, −0.05; B = 0.12, 95% CI = 0.09, 0.16, respectively).
- *Affirmation out-isolation concentration*: Women who had not previously sent an affirmation were less likely to send one (B = −0.34, 95% CI = −0.43, −0.24).
- *Affirmation same peer concentration*: Women who affirmed a peer were less likely to subsequently affirm or correct that peer (B = −0.15, 95% CI = −0.20, −0.10; B = −0.13, 95% CI = −0.20, −0.06, respectively).
- *Correction reciprocity*: Women tended to reciprocate corrections (B = 0.20, 95% CI = 0.13, 0.25).
- *Correction indegree activity*: Women tended to send corrections after receiving one (B = 0.13, 95% CI = 0.07, 0.19).
- *Correction indegree popularity*: Women tended to receive more corrections after receiving one (B = 0.24, 95% CI = 0.18, 0.30).
- *Correction outdegree activity*: Women tended to send more affirmations and corrections after sending a correction (B = 0.05, 95% CI = 0.02, 0.08; B = 0.06, 95% CI = 0.01, 0.10).
- *Correction out isolation activity*: Women who had not sent corrections at all were less likely to send a correction in subsequent periods (B = −0.34, 95% CI = −0.48, −0.20).
- *Correction same peer concentration*: Women who corrected a peer were more likely to subsequently correct the same peer (B = 0.16, 95% CI = 0.09, 0.23).
- *Time in TC activity and popularity*: Women were less likely to send either affirmations or corrections if they were in their first month of residence (B = −0.18, 95% CI = −0.24, −0.12; B = −0.18, 95% CI = −0.28, −0.08). They were more likely to receive affirmations if they were in their first month of residence (B = 0.11, 95% CI = 0.06, 0.16). They were less likely to send or receive corrections if they had been in the program for more than four months (B = −0.07, 95% CI = −0.14, −0.00; B = −0.09, 95% CI = −0.02, −0.17, respectively).
- *Admission time homophily*: Women were more likely to affirm and correct peers who arrived at about the same time (B = 0.23, 95% CI = 0.10, 0.37; B = 0.51, 95% CI = 0.29, 0.74, respectively).
- *Race/age/LSI-R activity/popularity*: European American residents were more likely to receive corrections (B = 0.29, 95% CI = 0.04, 0.58).
- *Race/age/LSI-R homophily*: Residents were more likely to affirm peers of approximately the same age (B = 0.19, 95% CI = 0.10, 0.27).
- *Staff affirmation indegree popularity*: Residents were less likely to receive an affirmation from peers after receiving one from staff than after receiving one from a peer (B = −0.04, 95% CI = −0.07, −0.00).
- *Staff correction indegree popularity*: Residents were less likely to receive a correction from peers after receiving one from staff than after receiving one from a peer (B = −0.04, 95% CI = −0.08, −0.01).
- *Head-turning indegree activity (corrections)*: Residents were less likely to send a correction after receiving a correction for head-turning (B = −0.52, 95% CI = −1.01, −0.07).
- *Staff head-turning indegree activity (corrections):* However, the coefficient that compares the result of receiving a head-turning correction from staff to the result of receiving one from peers is positive (B = 0.54, 95% CI = 0.03, 1.08). As the overall head-turning coefficient is negative and about the same size, this implies that following a peer head-turning correction the resident is less likely to correct a peer, whereas following a staff head-turning correction the change in likelihood is close to zero.

## Discussion

As a means of addressing the complexity of this model, we will consider the findings of the analysis under several broad headings.

### Residents primarily react to peers rather than their characteristics

The claim that mutual aid-based programs bring about change is based on an implicit prior claim that individuals who are involved in these programs react to each other. It is, therefore, encouraging to note that most of the statistically significant findings in this analysis involve peer interactions, with pre-existing characteristics of residents playing a comparatively minor role in network formation. Complex peer interactions have also been found among male TC residents (Doogan and Warren, 2017a; Doogan and Warren, 2017b). Yalom (1995) has identified the opportunity to exercise altruism as a cornerstone of group therapy, while Riessman (1965) has noted that those who help others often benefit themselves. The system of feedback in TCs gives plentiful opportunity for altruistic interactions.

### Therapeutic communities residents interact in ways that are known to promote cooperation

Studies of cooperation consistently show that direct reciprocity tends to maintain cooperation both in theoretical models and in experiments (Axelrod, 1984; Falk and Fischbacher, 2006; Rand and Nowak, 2013). It is not surprising that, like previous analyzes (Doogan and Warren, 2017a; Doogan and Warren, 2017b), this one shows reciprocity in both affirmations and corrections. TC clinical theory sees such reciprocal altruism, the exchange of helping behaviors, as helping residents to learn the value of sharing and helping others while reducing isolation (Frye, 1984; Stevens, 2013).

Generalized reciprocity also helps maintain cooperation in groups (Pfeiffer et al., 2005; Simpson et al., 2018; Stanca, 2009), as does reputation (Rand and Nowak, 2013). Residents are more likely to send both affirmations and corrections after receiving an affirmation, suggesting that generalized reciprocity plays a role in TCs and confirming the claim in De Leon (2000) that affirmations act to energize TC residents. This finding replicates the results of Doogan and Warren (2017a, 2017b) with male TC residents.

Receiving either an affirmation or correction from any peer correlates with an increased likelihood of subsequently receiving a peer correction. This suggests a reputation effect, which can benefit cooperation (Rand and Nowak, 2013). But it is not clear why both affirmations and corrections would increase the likelihood of receiving a correction; in the case of male TC residents, receiving affirmations increases the likelihood of receiving another affirmation, while receiving corrections increases the likelihood of receiving another correction (Doogan and Warren, 2017a; Doogan and Warren, 2017b). It is possible that for these female residents receiving either an affirmation or a correction calls attention to the individual, increasing the possibility of a subsequent correction (Mieth et al., 2017). This might also be a function of the small size of the unit. It is also possible that women are simply more willing to correct peers than men are. This finding raises the concern that corrections might be followed by further corrections, leading to a discouraging cycle (Doogan and Warren, 2017b). For women in this unit, receiving a correction soon after receiving an affirmation might act to reduce any encouragement from the affirmation (De Leon, 2000).

### Reaction to staff intervention is complex

Doogan and Warren (2017a, 2017b) found that peer affirmations increased the activity of male residents in sending affirmations and corrections but that staff affirmation and corrections did not. In this analysis, peer and staff affirmations lead to equivalent changes in resident activity. But staff affirmations and corrections both reduce the likelihood that residents will receive a correction more than peer affirmations, perhaps because peers see staff as assuming the role of giving feedback. Overall, this suggests that staff may have more leeway in directly intervening with female residents without reducing their activity in the TC, but that staff intervention may affect the resident network in other ways.

### Corrections for head-turning from peers are counterproductive

Head-turning corrections sanction residents who do not correct others. Contrary to our expectations, residents are less likely to send a correction after receiving a head-turning correction from a peer. No change in the number of peer corrections that residents send appears after receiving a head-turning correction from staff.

### These residents spread affirmations to the broad community

TC clinical theory suggests that residents should spread help across the entire community (De Leon, 2000). The negative coefficient on the same peer concentration for affirmations suggests that this does, indeed, happen in this unit – residents are less likely to affirm or correct a peer whom they have recently affirmed. Male residents, in contrast, are more likely to affirm residents whom they have recently affirmed (Doogan and Warren, 2017a). This finding offers quantitative confirmation of Mosher and Phillips’s (2006) finding that women in a TC extend their concern to the entire community and suggests that in this regard women might more easily adapt to TC treatment than men.

### Residents balance feedback to peers

Residents are more likely to send a correction after sending an affirmation and send an affirmation after sending a correction, similar to results found in Doogan and Warren (2017a, 2017b). This suggests that most residents try to engage in both aspects of the TC peer feedback system, possibly to manage their reputation as active members of the community.

### Isolation is self-sustaining

Residents who have not sent an affirmation or correction are less likely to send one, similar to the pattern observed in male TCs (Doogan and Warren, 2017a, 2017b). However, this result is specific to each network; that is, if a resident has not sent an affirmation, she is no more or less likely to send a correction and vice versa. Residents who are not participating in some part of the program may need intervention, either by staff or through the TC process of an encounter with peers (De Leon, 2000).

### There is a therapeutic community “life cycle:”

Women are most active, particularly in sending corrections, in the middle months of their residence. Men also show such a life cycle (Doogan and Warren, 2017a, 2017b). It is possible that residents move to means of supporting peers other than affirming and correcting them as they become more senior; for instance, more senior work positions involve mentoring peers and are often more time-consuming. It is also possible that the effective length of program treatment is less than the length of the program if residents decrease their activity toward the end of their stay.

Women also receive fewer corrections toward the end of treatment. A likely explanation for this is simply that they have learned TC norms of behavior. This is consistent with previous evidence that social learning occurs in TCs (Doogan and Warren, 2016; Warren, 2020).

### Homophily by entrance time occurs for both affirmations and corrections

Previous studies of male TC residents have found homophily by entrance time in both affirmations and corrections (Doogan and Warren, 2017a, 2017b), suggesting that residents build trust based on common experiences as they go through the TC. This study replicates that finding and suggests that entrance homophily might offer opportunities for clinicians to intervene early to foster cooperative relationships among TC residents. The only other source of homophily in this data set is age. In contrast with earlier analyses of male TCs (Doogan and Warren, 2017a), we found no evidence of homophily by race; this may be a result of the low percentage of minority women at this facility. However, this finding replicates a recent cross-sectional analysis that has also found that homophily by race at a women’s TC is nonsignificant (Cao et al., 2020) raising the possibility that homophily is less salient in TCs for women in general.

## Conclusion

This study is based on a small sample of women which is demographically atypical of the American correctional system as a whole (Kajstura, 2017). However, this analysis finds a set of relationships – reciprocity, generalized reciprocity, a willingness to spread affirmations and corrections, homophily by entrance time and balancing affirmative and corrective peer feedback – that together suggest that these women are interacting in ways that reinforce cooperation. In light of the evidence that unit atmosphere predicts outcomes (Carr and Ball, 2014) this is encouraging.

## Figures and Tables

**Table 1:** Latent space models of the exchange of affirmations and corrections in the TC for women

Variable	Affirmations model 95% CI				Corrections model 95% CI			
	Mean	Lower	Upper	Sig	Mean	Lower	Upper	Sig
Intercept	−1.83	−1.95	−1.71	*	−2.39	−2.49	−2.28	*
Affirmation reciprocity	0.14	0.10	0.18	*	−0.09	−0.15	−0.04	*
Affirmation transitive Closure	0.00	−0.03	0.02	*	0.00	−0.03	0.02	*
Affirmation three cycle	0.00	−0.01	0.01		0.02	0.00	0.03	*
Affirmation indegree activity	0.10	0.06	0.15	*	0.17	0.10	0.23	*
Affirmation indegree popularity	0.04	−0.01	0.09		0.14	0.05	0.22	*
Affirmation outdegree activity	−0.07	−0.10	−0.05	*	0.12	0.09	0.16	*
Affirmation out-isolation activity	−0.34	−0.43	−0.24	*	−0.09	−0.38	0.18	
Affirmation same peer concentration	−0.15	−0.20	−0.10	*	−0.13	−0.20	−0.06	*
Correction reciprocity	−0.05	−0.11	0.00		0.20	0.13	0.25	*
Correction transitive closure	0.02	−0.01	0.06		−0.04	−0.07	0.00	
Correction three cycles	0.03	0.00	0.07		−0.01	−0.04	0.03	
Correction indegree activity	0.03	0.00	0.07		0.13	0.07	0.19	*
Correction indegree popularity	0.02	−0.01	0.06		0.24	0.18	0.30	*
Correction outdegree activity	0.05	0.02	0.08	*	0.06	0.01	0.10	*
Correction out-isolation activity	−0.06	−0.12	0.01		−0.34	−0.48	−0.20	*
Correction same peer concentration	−0.05	−0.12	0.01		0.16	0.09	0.23	*
<1 month activity	−0.18	−0.24	−0.12	*	−0.18	−0.28	−0.08	*
<1 month popularity	0.11	0.06	0.16	*	−0.02	−0.10	0.06	
>4 months activity	0.04	−0.02	0.08		−0.07	−0.14	0.00	*
>4 months popularity	−0.01	−0.05	0.04		−0.09	−0.17	−0.02	*
Admission time homophily	0.23	0.10	0.37	*	0.51	0.29	0.74	*
White activity	0.27	−0.01	0.58		0.14	−0.04	0.33	
White popularity	−0.12	−0.26	0.01		0.29	0.04	0.58	*
Race homophily	0.05	−0.06	0.15		−0.08	−0.33	0.14	
Age activity	0.00	−0.01	0.01		−0.01	−0.02	0.00	
Age popularity	0.00	−0.01	0.00		0.00	−0.01	0.00	
Age homophily	0.19	0.10	0.27	*	−0.12	−0.26	0.01	
LSI-R activity	0.01	0.00	0.03		0.00	−0.01	0.01	
LSI-R popularity	0.00	0.00	0.01		0.00	0.00	0.01	
LSI-R similarity	0.00	−0.10	0.10		−0.03	−0.19	0.12	
Staff affirmation indegree-activity	0.02	−0.01	0.04		−0.03	−0.06	0.00	
Staff affirmation indegree-popularity	0.01	−0.02	0.03		−0.04	−0.07	0.00	*
Staff correction indegree-activity	−0.01	−0.04	0.02		−0.03	−0.06	0.01	
Staff correction indegree-popularity	0.00	−0.02	0.03		−0.04	−0.08	−0.01	*
Whole unit effect of HT	−0.12	−0.57	0.34		−0.15	−0.44	0.15	
Whole unit effect of staff HT	0.13	−0.33	0.59		0.15	−0.15	0.44	
HT indegree activity	0.24	−0.09	0.54		−0.52	−1.01	−0.07	*
Staff HT indegree activity	−0.35	−0.70	0.02		0.54	0.03	1.08	*
HT outdegree popularity	0.07	−0.28	0.40		−0.03	−0.46	0.39	
HT reciprocity	−0.17	−1.50	1.08		−0.90	−2.67	0.67	
Sender variance component	0.30	0.24	0.38	*	0.17	0.12	0.22	*
Receiver variance component	0.08	0.05	0.11	*	0.13	0.09	0.18	*
Day variance component	0.74	0.66	0.81	*	0.54	0.49	0.61	*

**Note:** * *p* <0.05
